# The Evolution of Total Phenolic Compounds and Antioxidant Activities during Ripening of Grapes (*Vitis vinifera* L., cv. Tempranillo) Grown in Semiarid Region: Effects of Cluster Thinning and Water Deficit

**DOI:** 10.3390/ijms17111923

**Published:** 2016-11-17

**Authors:** Inmaculada Garrido, David Uriarte, Marcos Hernández, José Luis Llerena, María Esperanza Valdés, Francisco Espinosa

**Affiliations:** 1 Department of Plant Biology, Ecology and Earth Sciences, University of Extremadura, 06006 Badajoz, Spain; igarridoc@unex.es (I.G.); jllerena@ctaex.com (J.L.L.); 2 CICYTEX-Institute of Agricultural Research Finca La Orden-Valdesequera, Ctra. A-V, Km 372, 06187 Badajoz, Spain; david.uriarte@gobex.es; 3 Aula Dei Scientific Technological Park Foundation, Av. Montañana 930, 50059 Zaragoza, Spain; marcoshsuarez@gmail.com; 4 Agri-Food Technological Center of Extremadura-CTAEX, 06195 Badajoz, Spain; 5 CICYTEX-Technological Institute of Food and Agriculture-INTAEX, Av. Adolfo Suárez s/n, 06071 Badajoz, Spain; esperanza.valdes@gobex.es

**Keywords:** antioxidant activities, irrigation, phenols, thinning, *Vitis vinifera*

## Abstract

A study was made of how water status (rainfed vs. irrigated) and crop load (no cluster thinning vs. cluster thinning) can together affect the grapes of *Vitis vinifera* cv. Tempranillo vines growing in a semiarid zone of Extremadura (Spain). The grapes were monitored at different stages of ripening, measuring the peroxidase (POX) and superoxide dismutase (SOD) antioxidant activities and the phenolic content (flavonoids and phenylpropanoids), together with other parameters. The irrigation regime was adjusted to provide 100% of crop evapotranspiration (ETc). The findings confirmed previous results that both thinning and water deficit advance ripening, while irrigation and high crop load (no thinning) lengthen the growth cycle. The SOD activity remained practically constant throughout ripening in the thinned treatments and was always lower than in the unthinned treatments, an aspect which could have been the cause of the observed greater level of lipid peroxidation in the water deficit, thinned treatment. The nonspecific peroxidase activity was very low, especially in the thinned treatments. The effect of thinning was enhanced when combined with water deficit, inducing increases in phenylpropanoids and, above all, flavonoids at the harvest stage of ripening, while leaving the polyphenol oxidase activity (PPO) unaffected.

## 1. Introduction

There have been numerous studies of the influence that viticulture techniques such as defoliation and thinning and the water management regime have on the quality of the grapes and the wines obtained with them. The precise answer has been found to depend largely on genotype [1,2] and soil and climate [3,4]. Thinning is a common viticulture practice designed to control yield and ripening under adverse conditions of climate. There is evidence that this practice improves the quality of the grapes [5,6]. Thus, this treatment has been described as inducing increases in total soluble solids [7,8] and in the anthocyanin and phenolic content [9,10,11]. In contrast, other studies have observed no change whatsoever in either the ripening period or the quality of the grapes [12]. The effect of thinning has also been described as depending on when [13] and how intensely [14] it is carried out. Uriarte et al. [15] and Valdés et al. [7] found that cluster thinning accelerates ripening without affecting vegetative growth or the grapes. This disparity of results has aroused some controversy as to the usefulness and profitability of this practice [16,17].

The application of water in irrigation treatments can also affect the plant’s physiology and its growth cycle, and consequently the quality of the grapes. It has been shown that a degree of water stress leads to improved grape quality [18]. Thus, in cv. Tempranillo and Chardonnnay vineyards (located in Madrid-Spain and Niagara Peninsula, Ontario, CA, USA), deficit irrigation (i.e., application of water in amounts less than 100% of crop evapotranspiration (ETc)), gave better grape quality than irrigation without water deficit [19,20]. In full agreement with those results, for the cv. Tempranillo grown in Extremadura, Valdés et al. [7] observed increases in the phenolic content of grapes and wines from vines irrigated at 25% ETc relative to those irrigated at 100% ETc.

Moreover, the water status of the vines may affect their response to different viticulture techniques. In this regard, there have been interesting studies of the different amplitude and significance of the effect that thinning has on the physical, chemical, and sensorial characteristics of grapes and wines from different areas in Spain [21,22,23,24].

Reactive oxygen species (ROS) have been implicated in plant growth and stress responses. The production and detoxification of ROS are both highly regulated processes, and ROS levels are kept under tight control. The antioxidant system plays an important part in ROS homeostasis [25,26]. It includes such enzymes as peroxidase (POX), catalase (CAT), and superoxide dismutase (SOD). Stresses and physiological processes in plants both induce the development of a complex network of oxidant/antioxidant reactions that modulate the resulting oxidative response [27,28]. Thus, a key role in the response to stresses is played by the activities involved in ROS production and elimination, and they are also involved in such physiological processes as ripening [29]. In this regard, while the participation of peroxidases, catalases, and superoxide dismutase is crucial for the control of the production or elimination of ROS, their activities are affected by both water stress and defoliation [30].

In plants, increased synthesis of phenols is a common response to stress [31], and the same environmental conditions that cause oxidative stress are associated with the induction of phenylpropanoid metabolism. Phenylpropanoids and flavonoids are involved in the protection against oxidative stress [32]. The level of lipid peroxidation and how the total antioxidant capacity and the phenolic compound content evolve can both be used as indicators of stress and the response to it. One response to stressors during ripening is an increase in the amount of phenolic compounds. These include flavonoids and phenylpropanoid glycosides [31,33]. The latter form part of the cell’s antioxidant system [34], and, together with flavonoids, participate in protecting against oxidative stress by eliminating ROS [32].

Ripening involves changes in the composition and accumulation of phenolic substances in the grapes, substances whose later extraction and transfer to the wine is essential. Antioxidant activity in grapes is positively correlated with the concentration of the phenols they contain [35], and the oxidation of compounds during ripening [36] may be due to enzymatic changes, as with the oxidation of phenols by phenol oxidases [37]. Alteration of the phenolic composition, polyphenol oxidase activity, and lipid peroxidation in response to the stress induced by defoliation has been shown in the Tempranillo variety [30]. Pastore et al. [38] studied the effect of defoliation on grapes of the Sangiovese variety at the transcriptional level. They found that the time at which the defoliation is performed alters the expression of such genes as those related to stress responses (ascorbate peroxidase and superoxide dismutase) and to flavonoid and phenylpropanoid synthesis.

Given this context, the objective of the present work was to study the evolution of some antioxidant activities such those of peroxidases and superoxide dismutase, the content of phenolic compounds (flavonoids, phenylpropanoids), and other parameters in response to drought and cluster thinning as factors that can induce stress in the vine. This stress in turn could influence the process of ripening of the grapes, altering the levels of ROS and phenols.

## 2. Results and Discussion

Large differences were observed in vine water status between irrigation treatments throughout the growing season (the stem water potential was −0.55 and −1.03 MPa in two and one treatments, respectively, see Material and Methods; *p* < 0.001). However, neither vine water status nor vine growth were affected by cluster thinning.

**Figure 1:**
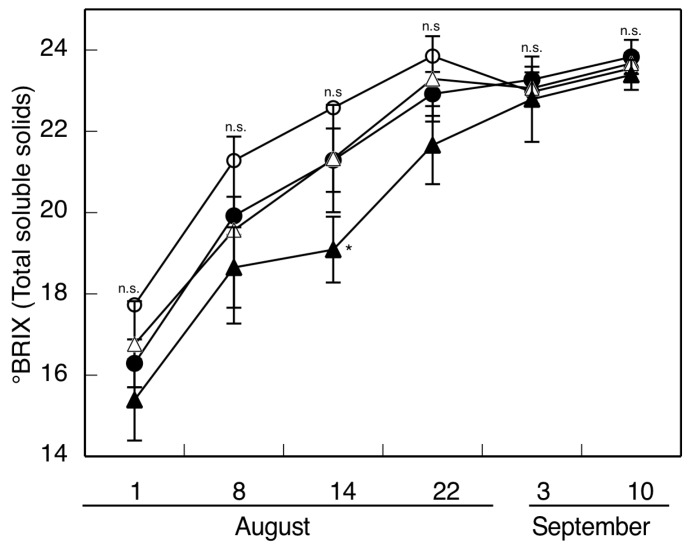
Evolution of TSS following veraison (in days). Treatments: (●) rainfed without thinning (H1), (○) rainfed with thinning (L1), (▲) irrigated without thinning (H2), and (△) irrigated with thinning (L2). The data are the means ± SD of at least 10 replicates obtained from 5 different experiments. According to Friedman test, significant differences (*p* < 0.05) are indicated by * and no significant differences are indicated as n.s.

Figure 1 shows the evolution of total soluble solids (TSS, °Brix) in the berries of the different treatments. One can deduce from the figure that the greater water stress supported by the rainfed than the irrigated vines increased the TSS of the grapes. Thinning had the same effect. Hence, in the L1 treatment (thinning in conditions of water stress), there was a considerable advance in the date of ripeness. The grapes at harvesting (S3) showed no statistically significant differences between irrigation and thinning treatments on the pH. The pH value was 3.8 for all treatments. The irrigation and cluster thinning led to statistically significant higher values of titratable acid (3.73c, 4.27bc, 5.93a, 5.34ab, for H1, L1, H2, and L2, respectively; see Material and Methods). These results are consistent with those reported by other workers [13,14,39]. Santesteban et al. [21] found for the Tempranillo variety that the main effect of thinning is the increase in TSS, and this increase depends on the intensity of the treatment. In this same sense, Diago et al. [2] observed for Garnacha that there are differences in °Brix at the same date of collection only when the berries come from intense thinning treatments, with those berries having a greater soluble solids content. Tardáguila et al. [17,40], however, reported increases in °Brix following thinning in one year but not the next. Likewise, Keller et al. [12] found thinning to have little effect on the growth cycle, ripening, or composition of the fruit of Cabernet Sauvignon, Riesling, and Chenin Blanc vines, with the environmental conditions having a clearly greater influence.

**Figure 2:**
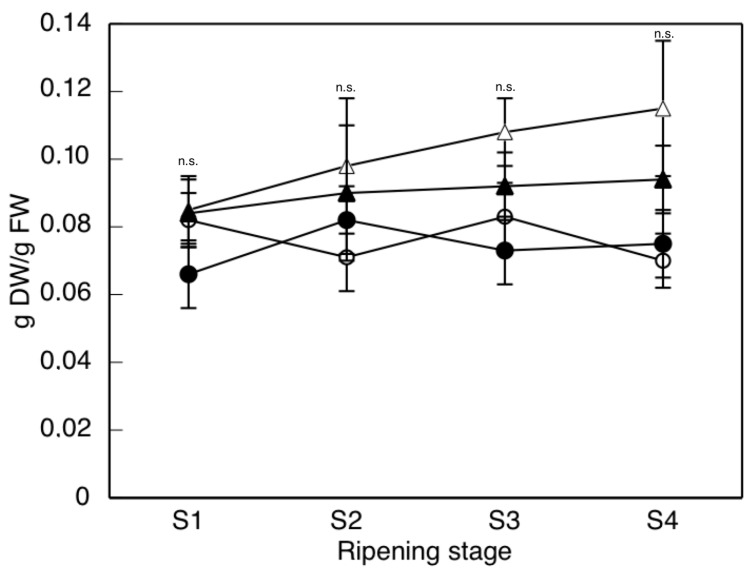
The DW/FW ratio at different stages of ripening. Treatments: (●) rainfed without thinning (H1), (○) rainfed with thinning (L1), (▲) irrigated without thinning (H2), and (△) irrigated with thinning (L2). The data are the means ± SD of at least 10 replicates obtained from 5 different experiments. According to Friedman test, no significant differences are indicated by n.s.

With respect to the dry weight to fresh weight (DW/FW) ratio of the berries (Figure 2), in the cases with water restriction (H1 and L1) there was little change over the course of ripening, and the values were always below those corresponding to L2, an effect similar to that described by Girona et al. [41] working with the same variety. Thinning affected this ratio only in the L2 grapes, causing a gradual increase in this parameter so that, at stages S3 and S4, the values found in them were manifestly greater than in the rest. Thinning increases dry weight by modifying the source/sink ratio, leading to a greater supply of nutrients [42].

One observes in Table 1 that the evolution of the phenol and the phenylpropanoid contents was similar, and that it was modified by the treatments relative to the control values. Total phenols in the unthinned cases (H1 and H2) showed the same pattern of evolution—a gradual rise from S2 onwards to final values very close to the initial measurements. No clear trend was observed when considering the fluctuations. Thinning altered this behavior, but in different ways depending on the water status. For L1, there were very high values of the total phenols and the phenylpropanoid contents at S1, followed by a sharp drop at S2, and then relatively little change through to S4. No such drop was observed for L2, however, in which the values were very similar throughout ripening, and below those of L1 in all the samples except for S4 in which they were slightly higher.

The evolution of the flavonoids was similar to that of the phenols and phenylpropanoid glycosides in the H1, H2, and L2 grapes. In L1, however, fluctuations were observed throughout the cycle. These results indicate firstly that, in terms of techniques, all the compounds analyzed were more affected by thinning than by water stress. The combination of the two treatments induced a strong increase in all three families of compounds at S1, but at harvest (S3) their levels were similar to the control values, with the exception of the flavonoids. Secondly, the effect was in general greater on the phenylpropanoids and flavonoids than on the total phenols.

With thinning, all these phenolic components were found in higher or equal concentrations, but not lower. This is consistent with the results of Tardáguila et al. [40] who describe increased overall amounts of phenols and anthocyanins in grapes from thinned cv. Tempranillo vines. Likewise, Diago et al. [2] also describe an increase in these compounds caused by thinning in this variety, although the treatment was much less effective in the Garnacha variety. Guidoni et al. [9] found cluster thinning to increase berry-skin flavonoids, with the effect being relatively larger in anthocyanins in Nebbiolo berries. They report water deficit as having a similar effect, with the phenol content over the course of ripening generally being greater or the same when the plants are not irrigated. Esteban et al. [19] and Ojeda et al. [43] describe similar results in the content of total phenols, flavonoids, anthocyanins, and flavonols as a result of water stress. Again, the combination of water deficit and thinning is the treatment with the greatest incidence on the evolution and content of these phenolic compounds. At harvest, however, the highest values correspond to the irrigated treatments. In a trial similar to the present study in the same experimental vineyard, both Gamero et al. [23] and Intrigliolo and Castela [44] found increases in anthocyanins in wines from vines irrigated with more water. The researchers explained these findings on the basis of the greater water needs and lack of adaptation of cv. Tempranillo (a variety typical of cooler zones) to the soil and climate conditions of the vineyards. A further contributing factor could have been better synchrony in the ripening of the pulp and the skins in the irrigated grapes.

In Table 2, one can observe how membrane lipid peroxidation evolves as an indicator of oxidative stress. In all the treatments, there is an increase from S1 to S2 which is of greater amplitude in the rainfed treatments. It is noteworthy that, throughout the ripening process, the greatest values of lipid peroxidation correspond to L1 (rainfed and thinned). Various authors have reported an increase in lipid peroxidation during fruit ripening. Yildirim et al. [45], for example, describe such an increase from veraison to ripeness of grapes, and Rogiers et al. [46] describe this increase in the ripening of Saskatoon fruit. This increase in the levels of lipid peroxidation is indicative of an increase in oxidative stress during the development of the fruit, to which would have to be added that, in the L1 treatment (with the highest values), the lack of irrigation and the cluster thinning could cause the plant greater oxidative stress.

The SOD activity remained practically constant throughout ripening in the thinned treatments, and was always lower than in the unthinned treatments. In these latter (H1 and H2), different trends were observed. While in the H1 grapes there was a steady increase in SOD, in the H2 grapes, after an initial increase and then stabilization, there was a decline in the last sample. It stands out that the lowest values of SOD activity corresponded to L1. This would be consistent with the fact that the highest values of lipid peroxidation were found for this treatment since the low SOD activity could lead to an increase in the ROS responsible for lipid peroxidation, so that this is the treatment that causes the greatest oxidative stress.

Regarding the activity of PPO, an enzyme which catalyzes the formation of o-quinones, at S2 it was increased by thinning (both rainfed and irrigated), possibly as a consequence of the higher levels of phenols obtained with this technique. However, at harvest (S3), the PPO activity levels were similar in all treatments. At S2, the effect of thinning on this activity is the opposite to that of defoliation which induces a decrease in PPO activity, and hence in the possibility of phenol oxidation [30]. Negri et al. [47] observe maximum PPO expression at veraison, followed by a decline with ripening, as was the case in the present study. Thinning appears to affect PPO activity more than water stress since, in the thinning treatments, PPO activity levels remained higher for longer. The greater PPO activity indicates that, at S2, there is increased polyphenol oxidation. Nevertheless, that this activity decreases to values similar to the controls at S3 reduces the risk of alteration of phenolic compounds and the effect it would have on the resulting wines. This evolution allows thinning to be applied without it altering the levels of polyphenol oxidation.

The nonspecific POX activity levels were generally very low, declining from S1 to S4 (except for H2). The lowest values correspond to the thinning treatments. Yildirim et al. [45] report declining glutathione peroxidase activity from veraison to ripeness. Our results for this activity could explain those we obtained for lipid peroxidation, since the greater the POX activity (more consumption of H_2_O_2_), the lesser the lipid peroxidation.

The activity of CA-POX, an enzyme which is involved in processes of lignification, showed oscillations in all treatments. While thinning led to an increase in this activity at S2 in the rainfed case, it led to a decrease in the irrigation case. At S3 (harvest), this activity's levels were slightly higher with thinning than without thinning. This enzyme might be involved in the production of H_2_O_2_ during the formation of lignins [48]. According to our results, it does not appear to be affected by either the stage of ripening or the different treatments (thinning and water deficit). At the transcriptome level, defoliation alters its expression depending on when the technique is applied [38], so that, perhaps in our case too, thinning could influence this activity depending on the timing of its application.

Taken together, our results indicate that both thinning and water deficit induce an advance in ripening, while irrigation and high crop load (no thinning) lead to a longer growth cycle, confirming previous results [7]. The effect of thinning is enhanced when combined with water deficit, inducing increases in phenylpropanoids and especially flavonoids at the time of harvest, without the PPO being affected at that stage of ripening.

## 3. Material and Methods

### 3.1. Plant Material and Experimental Design

The experiment was carried out during the 2012 season in Extremadura (which has a Mediterranean climate) in a vineyard of the Tempranillo (*Vitis vinifera* L.) variety, planted in 2001 on Richter-110 rootstock at a spacing of 2.5 m by 1.2 m (3333 vines·ha^−1^). The vineyard is located at Finca la Orden (Regional Government of Extremadura, Badajoz, Spain). Vines were trained to a vertical trellis in a bilateral cordon system with east-west orientation. Winter pruning was to six spurs per vine, two buds per spur. The soil at the site has a loam to clay-loam texture. The volumetric water content is 20.4% at field capacity and 11.4% at permanent wilting point. In 2012, total rainfall during the growth cycle was 136.20 mm, average temperature 20.82 °C, average relative humidity (HR) 55.60%, and solar radiation 23.87 MJ·m^−2^·day^−1^. The climatic conditions are summarized in Table 3.

The experimental design was split plot with four replicates. Irrigation was the whole plot factor, and cluster thinning the subplot factor. The experimental plots had six rows with eighteen vines per row. The irrigation treatments were 0% ETc (rainfed, non-irrigated vines, 1) and 100% ETc (irrigated vines, 2). The value of ETc was determined with a weighing lysimeter installed in the experimental vineyard [49]. Irrigation was started when the stem water potential (SWP) reached a level of −0.6 MPa, a threshold based on the studies of Williams and Trout [50] and Williams and Baeza [51], and was terminated at the end of September. The system applied was drip irrigation with pressure-compensated emitters of 4·L·h^−1^ located in a single row 120 cm apart. Within each irrigation regime, two crop yield levels were tested—unthinned (high crop yield, H) and thinned (low crop yield, L). Thinning was by hand at close to fruit set. One cluster was retained per shoot. The SWP measurements were made at midday using a pressure chamber (Model Soil Moisture Corp., Santa Barbara, CA, USA).

Following veraison, berry ripening was monitored fortnightly. Berries were sampled from 28 vines of each experimental plot (112 vines per treatment), taking them from all parts of the canopy for the sample to be as representative and homogeneous as possible.

The must total soluble solids content (TSS) was determined weekly. Berries were collected by removing small portions of clusters in the early morning, and each berry was separated from its pedicel in the laboratory. Samples of 250 g per plot were crushed, and the TSS (°Brix) was determined by refractometry (Total Atago RX-1000 refractometer, Tokyo, Japan). The criterion established for harvest was 23 °Brix (the common criterion for this variety in this area). At harvest, titratable acidity (TA) and pH were also determined following the official methods of the International Organization of Wine and Vine (OIV) [52]. An aliquot of this juice was immediately frozen and stored at −20 °C until assay. Grapes (5 g) were dried in a forced air oven at 70 °C for 24 h to obtain the dry weight (DW).

### 3.2. Biochemical Assay

For the biochemical assay, four ripening stages were used in accordance with the TSS: stage I (S1) (18.5 ± 0.5 °Brix); stage II (S2) (21.5 ± 0.5 °Brix); stage III or harvest (S3) (22.5 ± 0.5 °Brix); and stage IV or post-harvest (S4) (23.5 ± 0.5 °Brix).

Phenols, flavonoids, and phenylpropanoid glycosides were assayed colorimetrically. First, grapes were homogenized with methanol, chloroform, and 1% NaCl (1:1:0.5). The homogenate was filtered and centrifuged at 3200× *g* for 10 min. Total phenols (expressed as µg caffeic acid g^−1^·FW) were determined at 765 nm with Folin-Ciocalteu reagent according to the method of Singleton et al. [53]. Total flavonoids (expressed as µg rutin^−1^·FW) were determined according to the method of Kim et al. [54], calculating the content on the basis of the rutin standard curve. Phenylpropanoids (expressed as µg verbascoside g^−1^·FW) were determined at 525 nm based on estimating an *O*-dihydroxycinnamic derivative using the Arnow reagent as described in Gálvez et al. [55], calculating the content on the basis of the 3,4-dihydroxyphenylalanine standard curve.

Lipid peroxidation was determined by measuring malondialdehyde (MDA) formation using the thiobarbituric acid (TBA) method as described by Madhava Rao and Sresty [56]. The MDA concentration (expressed as nmol MDA g^−1^·FW) was calculated using an extinction coefficient of *ε* = 155 mM^−1^·cm^−1^.

### 3.3. Enzymatic Activities

Enzymatic activities were determined on a crude extract of the grapes. The grapes (2 g·mL^−1^) were homogenized at 4 °C in 50 mM phosphate buffer, pH 6.0. The homogenate was filtered and centrifuged at 39,000× *g* for 30 min at 4 °C. The pellet was discarded, and the supernatant filtered and collected for the enzyme assays. The protein content was determined by the method of Bradford [57].

Peroxidase (EC 1.11.1.7) activity, POX, was measured at 590 nm (*ε* = 47.6 mM^−1^·cm^−1^) [58], with the reaction medium consisting of the enzyme extract, 3.3 mM DMAB, and 66.6 µM MBTH in 50 mM phosphate buffer, pH 6.0. A unit of POX is defined as the amount of enzyme required to cause the formation of 1 nmol DMAB-MBTH (indamine dye) per minute at 25 °C, pH 6.0. The coniferyl alcohol (CA) peroxidase activity, CA-POX, was determined by measuring the decrease in absorbance at 265 nm of a reaction medium consisting of the enzyme extract and 0.1 mM CA in 25 mM acetate buffer pH 5.0 (*ε* = 7.5 mM^−1^·cm^−1^). A unit of CA-POX is defined as the amount of enzyme required to cause the oxidation of 1 nmol CA per minute at 25 °C, pH 5.0.

Superoxide dismutase (EC 1.15.1.1) activity, SOD, was determined from the absorbance at 560 nm of the enzyme extract in 50 mM phosphate buffer pH 7.8, 0.1 mM EDTA, 1.3 µM riboflavin, 13 mM methionine, and 63 µM NBT [59]. A unit of SOD is defined as the amount of enzyme required to cause 50% inhibition of NBT reduction.

Polyphenol oxidase (EC 1.14.18.1) activity, PPO, was determined from the absorbance at 390 nm at 30 °C of a reaction medium consisting of the enzyme extract, 100 mM phosphate buffer, Triton X-100, and 30 µM caffeic acid [60]. A unit of PPO is defined as the amount of enzyme required to cause a decrease in absorption of 0.001 units·min^−1^.

### 3.4. Statistical Analyses

The data are presented as the means ± SD of at least 10 replicates obtained from 5 independent experiments. The Friedman test was used to compare sample means, taking *p* < 0.05 as the significance level. These statistical calculations were performed using the SPSS version 21.0 program package (SPSS Inc., Chicago, IL, USA).

## Figures and Tables

**Table 1:** Total phenols, flavonoids, and phenylpropanoid glycosides in the different treatments and ripening stages used. Data are the means ± SD of at least 10 replicates obtained from 5 different experiments. The values were subjected to the Friedman test.

Treatment	Ripening Stage	Total Phenols (µg Caffeic Acid g^−1^ FW)	Phenylpropanoid Glycosides (µg Verbascoside g^−1^ FW)	Total Flavonoids (µg Rutin g^−1^ FW)
Rainfed unthinned (H1)	S1	919.2 ± 177.0 ^ab^	2075.1 ± 212.6 ^b^	1133.9 ± 206.3 ^b^
	S2	757.7 ± 64.1 ^a^	1356.6 ± 147.0 ^a^	788.6 ± 80.0 ^a^
	S3	838.0 ± 136.0 ^ab^	1450.8 ± 175.5 ^a^	1139.3 ± 208.1 ^b^
	S4	997.6 ± 161.0 ^b^	1735.7 ± 141.3 ^ab^	1519.0 ± 286.5 ^b^
Irrigated unthinned (H2)	S1	810.7 ± 189.0 ^b^	1548.8 ± 290.0	803.2 ± 256.3 ^b^
	S2	556.4 ± 49.4 ^a^	1512.6 ± 201.0	577.0 ± 38.1 ^a^
	S3	928.6 ± 99.6 ^b^	1673.9 ± 253.4	1231.1 ± 148.6 ^c^
	S4	1038.9 ± 191.3 ^b^	1842.2 ± 324.9	1489.6 ± 401.0 ^c^
Rainfed and thinned (L1)	S1	1558.4 ± 172.5 ^b^	2504.3 ± 218.0 ^c^	1688.2 ± 319.5 ^b^
	S2	1052.7 ± 128.4 ^a^	1965.1 ± 303.5 ^b^	1223.2 ± 101.0 ^a^
	S3	1010.7 ± 174.5 ^a^	1870.6 ± 275.6 ^ab^	1531.5 ± 129.6 ^b^
	S4	953.5 ± 59.4 ^a^	1541.1 ± 113.5 ^a^	1179.8 ± 124.5 ^a^
Irrigated and thinned (L2)	S1	804.2 ± 169.0	1624.5 ± 425.0	927.4 ± 216.4 ^a^
	S2	1035.2 ± 177.5	1803.6 ± 316.4	1141.6 ± 202.8 ^ab^
	S3	939.0 ± 158.0	1735.0 ± 124.3	1168.4 ± 246.0 ^ab^
	S4	1115.4 ± 146.0	1865.6 ± 6.3	1502.8 ± 167.6 ^b^

Different letters within each column by treatment indicate significant difference according to Friedman test (*p* = 0.05).

**Table 2:** Lipid peroxidation, and the polyphenol oxidase (PPO), superoxide dismutase (SOD), nonspecific peroxidase (POX), and coniferyl alcohol peroxidase (CA-POX) activities in the different treatments and ripening stages used. Data are the means ± SD of at least 10 replicates obtained from 5 different experiments. The values were subjected to the Friedman test.

Treatment	Ripening Stage	Lipid Peroxidation (nmol MDA g^−1^ FW)	PPO (UPPO mg^−1^ Protein)	SOD (USOD mg^−1^ Protein)	POX (UPOX mg^−1^ Protein)	CA-POX (UCA-POX mg^−1^ Protein)
Rainfed unthinned (H1)	S1	47.2 ± 14.9 ^a^	3143.2 ± 325.0 ^c^	62.7 ± 5.6	9.4 ± 3.1 ^b^	53.9 ± 15.0
	S2	79.5 ± 13.5 ^b^	253.7 ± 37.3 ^a^	76.9 ± 9.8	3.7 ± 0.9 ^a^	28.6 ± 8.7
	S3	86.6 ± 21.9 ^b^	1314.5 ± 682.0 ^b^	86.5 ± 20.1	4.6 ± 1.0 ^a^	50.3 ± 17.3
	S4	75.6 ± 9.1 ^b^	921.5 ± 42.0 ^b^	104.9 ± 38.5	3.4 ± 1.0 ^a^	62.9 ± 9.7
Irrigated unthinned (H2)	S1	46.2 ± 14.2 ^a^	1862.8 ± 402.3 ^c^	103.6 ± 25.9 ^a^	4.9 ± 1.5 ^b^	69.9 ± 18.3
	S2	80.1 ± 16.0 ^b^	315.2 ± 136.0 ^a^	141.9 ± 17.2 ^b^	6.6 ± 1.9 ^b^	67.8 ± 15.1
	S3	71.3 ± 15.8 ^ab^	1363.3 ± 184.0 ^c^	142.3 ± 27.9 ^b^	4.4 ± 0.7 ^b^	70.4 ± 12.4
	S4	93.7 ± 2.9 ^b^	656.9 ± 166.0 ^b^	103.6 ± 4.2 ^a^	2.0 ± 0.1 ^a^	45.7 ± 15.6
Rainfed thinned (L1)	S1	71.0 ± 34.0 ^a^	2461.8 ± 497.0 ^b^	49.5 ± 7.6	5.1 ± 0.6 ^b^	61.4 ± 19.0
	S2	130.3 ± 19.1 ^b^	2704.0 ± 358.0 ^b^	62.3 ± 19.0	2.1 ± 0.3 ^a^	72.5 ± 18.9
	S3	121.4 ± 26.2 ^ab^	805.4 ± 224.2 ^a^	65.0 ± 15.7	3.8 ± 1.2 ^ab^	61.7 ± 14.7
	S4	98.8 ± 9.2 ^a^	1471.5 ± 697.3 ^ab^	47.8 ± 1.1	3.0 ± 0.6 ^a^	51.8 ± 19.8
Irrigated thinned (L2)	S1	39.9 ± 21.5 ^a^	2322.4 ± 328.5 ^b^	94.9 ± 5.0	5.6 ± 1.3 ^b^	71.9 ± 28.6
	S2	72.4 ± 20.0 ^ab^	2992.5 ± 130.0 ^c^	76.1 ± 25.4	2.3 ± 1.1 ^a^	44.1 ± 6.5
	S3	78.6 ± 20.4 ^ab^	1134.3 ± 282.4 ^a^	80.7 ± 18.2	2.7 ± 0.9 ^a^	72.4 ± 26.1
	S4	97.9 ± 19.6 ^b^	1273.5 ± 329.0 ^a^	73.8 ± 15.5	1.7 ± 0.5 ^a^	52.7 ± 8.0

Different letters within each column by treatment indicate significant difference according to Friedman test (*p* = 0.05).

**Table 3:** Environmental conditions during the 2012 growth cycle.

Month	Rainfall (mm)	T_max_ (°C)	T_min_ (°C)	T (°C)	% HR _max_	% HR _min_	% HR	Solar Radiation (MJ·m^−2^·day^−1^)	Net Solar Radiation (MJ·m^−2^·day^−1^)
January	14.80	14.31	−0.47	5.88	83.96	52.68	81.47	9.34	2.43
February	2.80	15.63	−3.06	5.93	85.32	24.79	55.95	14.81	4.58
March	6.40	20.99	4.43	12.75	79.75	24.27	51.11	17.74	7.70
April	50.60	18.41	7.46	12.71	87.61	40.57	66.81	17.60	9.68
May	37.80	27.50	12.26	19.91	86.28	27.70	57.60	24.35	13.45
June	0.00	30.54	14.68	22.67	82.79	22.90	52.43	28.12	15.52
July	0.00	32.78	14.96	23.93	79.75	19.39	48.70	29.51	15.72
August	0.20	32.56	15.00	24.20	83.40	22.25	51.27	25.38	13.19
September	47.60	29.24	13.75	21.49	83.87	29.48	56.80	18.25	8.89
October	62.20	23.03	10.58	16.48	91.49	42.64	72.56	13.05	5.52
November	98.00	17.03	7.68	12.08	94.23	59.38	81.37	7.99	2.86
December	58.00	13.70	4.56	8.70	98.50	72.30	90.88	6.12	1.98
Annual	378.40	22.98	8.49	15.56	87.25	36.53	63.92	17.69	8.46
Growth cycle	136.20	28.51	13.02	20.82	83.95	27.05	55.60	23.87	12.74
